# Supplementary material for: Mechanisms governing the pioneering and redistribution capabilities of the non-classical pioneer PU.1
Source: Nat Commun. 2020 Jan 21;11:402. doi: 10.1038/s41467-019-13960-2 (PMC6972792; doi:10.1038/s41467-019-13960-2)
Supplement: Supplementary file 7 — Source data [file 41467_2019_13960_MOESM7_ESM.zip › Source_Data/Figure6/Figure6B_MotifScanOutput/FLI1_mutPU.1/homerResults/motif1.similar.html]

motif1

## Information for motif1

C
T
G
A
T
A
G
C
T
G
A
C
C
T
A
G
T
A
C
G
C
T
G
A
C
G
T
A
T
C
A
G
A
G
C
T
T
A
C
G
T
C
A
G
  
Reverse Opposite:  

A
G
T
C
A
T
G
C
T
C
G
A
A
G
T
C
G
C
A
T
G
A
C
T
A
T
G
C
G
A
T
C
A
C
T
G
A
T
C
G
G
A
C
T
  

|  |  |
| --- | --- |
| p-value: | 1e-3291 |
| log p-value: | -7.578e+03 |
| Information Content per bp: | 1.577 |
| Number of Target Sequences with motif | 8765.0 |
| Percentage of Target Sequences with motif | 85.43% |
| Number of Background Sequences with motif | 7713.5 |
| Percentage of Background Sequences with motif | 20.24% |
| Average Position of motif in Targets | 101.4 +/- 40.8bp |
| Average Position of motif in Background | 99.1 +/- 65.4bp |
| Strand Bias (log2 ratio + to - strand density) | 0.0 |
| Multiplicity (# of sites on avg that occur together) | 1.52 |
| Motif File: | file (matrix) reverse opposite |

### Similar de novo motifs found

|  |  |  |  |  |  |  |  |
| --- | --- | --- | --- | --- | --- | --- | --- |
| Rank | Match Score | Redundant Motif | P-value | log P-value | % of Targets | % of Background | Motif file |
| 1 | 0.995 | T A C G A G T C A T G C T C G A A G T C G C A T A C G T A T G C G A T C A C T G A T C G G A C T | 1e-3226 | -7429.908890 | 85.35% | 20.72% | motif file (matrix) |
| 2 | 0.977 | A T G C T C A G A G T C C G A T A C G T A G T C A G T C A C T G A C T G G A C T | 1e-3205 | -7382.125826 | 86.15% | 21.65% | motif file (matrix) |
| 3 | 0.986 | T C A G A G T C C G A T C G A T A G T C G A T C A C T G A T C G G A C T | 1e-3193 | -7352.295982 | 85.19% | 20.86% | motif file (matrix) |
| 4 | 0.980 | T C A G A G T C C G A T A C G T A G T C G T A C A C T G A T C G | 1e-2935 | -6758.369881 | 88.22% | 26.33% | motif file (matrix) |
| 5 | 0.987 | G T C A T A C G A G T C A G T C T C G A A G T C C G A T A C G T A G T C A G T C A C G T A T C G G A C T | 1e-2895 | -6667.863315 | 83.86% | 22.30% | motif file (matrix) |
| 6 | 0.946 | C T A G A G T C C G A T A C G T A G T C G T A C A C T G | 1e-2647 | -6097.201779 | 89.26% | 30.58% | motif file (matrix) |
| 7 | 0.976 | T C A G T C A G C T G A T A G C T G C A A C T G A C T G C G T A C G T A T C A G A G C T T C A G T C A G T A G C | 1e-2504 | -5766.225741 | 83.24% | 25.60% | motif file (matrix) |
| 8 | 0.914 | T G A C C T A G A C T G C G T A G C T A T C A G G A C T C A T G C T A G | 1e-1715 | -3949.217515 | 58.05% | 13.70% | motif file (matrix) |
| 9 | 0.865 | C T G A A G T C G T C A A C T G A C T G C G T A C G T A | 1e-1610 | -3707.241031 | 56.69% | 13.83% | motif file (matrix) |
| 10 | 0.887 | C T G A T A G C T C G A A C T G A C T G C G T A G C T A T C A G G C A T | 1e-1429 | -3290.559857 | 44.39% | 8.29% | motif file (matrix) |
| 11 | 0.881 | A G T C C G A T A C G T A G T C G T A C A C T G A T C G A C G T | 1e-1398 | -3220.551721 | 46.70% | 9.80% | motif file (matrix) |
| 12 | 0.889 | T G A C C T A G A T C G C G T A C G T A T C G A C G A T T C A G | 1e-1275 | -2936.973223 | 51.00% | 13.61% | motif file (matrix) |
| 13 | 0.838 | G A T C G T A C T C G A A G T C C G A T A G C T A T G C A G T C A G C T A T C G | 1e-1083 | -2495.802205 | 36.60% | 7.18% | motif file (matrix) |
| 14 | 0.793 | T C G A A T G C A C G T A C G T T G A C A G T C A C G T T C G A G C A T | 1e-980 | -2258.519717 | 40.07% | 10.08% | motif file (matrix) |
| 15 | 0.805 | A C T G A C T G C G T A C G T A C T A G A C G T A C T G | 1e-945 | -2178.122103 | 38.04% | 9.28% | motif file (matrix) |
| 16 | 0.804 | A G T C A C G T A C G T A G T C A G T C A G C T A T G C | 1e-670 | -1543.520177 | 52.35% | 23.26% | motif file (matrix) |
| 17 | 0.848 | C G A T C G A T A T G C A G T C A C T G A C T G G T C A | 1e-568 | -1308.490764 | 41.19% | 16.51% | motif file (matrix) |
| 18 | 0.767 | T C G A C T A G A G C T C T G A A C T G A C T G C G T A C G T A A C T G A C G T C T A G T C A G A T G C | 1e-530 | -1220.767056 | 16.07% | 2.31% | motif file (matrix) |
| 19 | 0.845 | A C T G A T G C G T A C A C T G A C T G C G T A C G T A A C T G | 1e-450 | -1038.099416 | 19.38% | 4.51% | motif file (matrix) |
| 20 | 0.783 | A T C G C G T A A T C G A G T C C G A T A C G T A G T C A G T C A C G T T A C G | 1e-325 | -749.693315 | 23.54% | 8.65% | motif file (matrix) |
| 21 | 0.762 | T C G A T C A G C T G A T A G C G T C A C T A G A C T G C G T A A C G T A C T G A G C T A C T G C T A G | 1e-295 | -680.817272 | 12.78% | 2.88% | motif file (matrix) |
| 22 | 0.841 | A C G T A C G T A C G T A G T C A G T C A C T G A C T G | 1e-279 | -644.633859 | 9.29% | 1.47% | motif file (matrix) |
| 23 | 0.798 | T A G C A T G C A C T G C T A G G T C A C G T A T G C A T C G A G T C A | 1e-127 | -294.026800 | 6.60% | 1.73% | motif file (matrix) |
| 24 | 0.767 | T A G C T C A G A G T C A G C T G A C T A T G C G T A C C T A G A T C G T C G A G T C A T C G A T A G C T G A C | 1e-78 | -181.365949 | 4.18% | 1.12% | motif file (matrix) |
| 25 | 0.806 | A C T G C A T G T C A G C A G T A C G T A C G T G T A C A T G C C A T G C A T G T A C G A T C G T A G C | 1e-47 | -109.876350 | 2.16% | 0.50% | motif file (matrix) |
| 26 | 0.791 | A T C G C T A G C T A G C G A T A C G T A T G C A T G C A T C G A C T G C A G T A C G T A T C G | 1e-42 | -98.306537 | 3.31% | 1.19% | motif file (matrix) |
